# Health-Related Quality of Life in Relation to Obesity Grade, Type 2 Diabetes, Metabolic Syndrome and Inflammation

**DOI:** 10.1371/journal.pone.0140599

**Published:** 2015-10-16

**Authors:** Sandra N. Slagter, Jana V. van Vliet-Ostaptchouk, André P. van Beek, Joost C. Keers, Helen L. Lutgers, Melanie M. van der Klauw, Bruce H. R. Wolffenbuttel

**Affiliations:** 1 Department of Endocrinology, University of Groningen, University Medical Center Groningen, Groningen, The Netherlands; 2 Van Swieten Research Institute, Martini Hospital, Groningen, The Netherlands; Dasman Diabetes Institute, KUWAIT

## Abstract

**Background:**

Health-related quality of life (HR-QoL) may be compromised in obese individuals, depending on the presence of other complications. The aim of this study is to assess the effect of obesity-related conditions on HR-QoL. These conditions are i) grade of obesity with and without type 2 diabetes (T2D), ii) metabolic syndrome (MetS), and iii) level of inflammation.

**Methods:**

From the Dutch LifeLines Cohort Study we included 13,686 obese individuals, aged 18–80 years. HR-QoL was measured with the RAND 36-Item Health Survey which encompasses eight health domains. We calculated the percentage of obese individuals with poor HR-QoL, i.e. those scoring below the domain and sex specific cut-off value derived from the normal weight population. Logistic regression analysis was used to calculate the probability of having poor domain scores according to the conditions under study.

**Results:**

Higher grades of obesity and the additional presence of T2D were associated with lower HR-QoL, particularly in the domains physical functioning (men: odds ratios (ORs) 1.48–11.34, *P*<0.005, and women: ORs 1.66–5.05, *P*<0.001) and general health (men: ORs 1.44–3.07, *P*<0.005, and women: ORs 1.36–3.73, *P*<0.001). A higher percentage of obese individuals with MetS had a poor HR-QoL than those without MetS. Furthermore, we observed a linear trend between inflammation and the percentage of obese individuals with poor scores on the HR-QoL domains. Individuals with MetS were more likely to have poor scores in the domains general health, vitality, social functioning and role limitations due to emotional problems. Obese women with increased inflammation levels were more likely to have poor scores on all domains except role limitations due to emotional problems and mental health.

**Conclusions:**

The impact of obesity on an individual’s quality of life is enhanced by grade of obesity, T2D, MetS and inflammation and are mainly related to reduced physical health. The mental well-being is less often impaired.

## Introduction

A recent study across seven European countries estimated that obesity prevalence varies between 12% and 26% [1], confirming that obesity has become an epidemic [2]. However, the effect of obesity goes beyond obesity-related and increased morbidities and reduced life expectancy.

Impaired health-related quality of life (HR-QoL) has been found in individuals with obesity. Obesity has a greater impact on domains of physical health than on domains of mental well-being [3]. However, our current knowledge is limited as studies made no distinction between the grades of obesity [4–7], had small numbers of participants in the different grades of obesity [8–11] or examined only two groups of obesity [8, 11–13]. In addition, often self-reported height and weight were used to calculate the body mass index (BMI) [5, 8, 10, 12, 14]. Other issues that arise with the previously published studies is the wide use of different instruments to assess HR-QoL [4, 7, 13, 14] and taking no morbidities into account, or only a small subset of morbidities [5, 6, 13, 14]. Next, obesity often coincides with metabolic syndrome (MetS), type 2 diabetes (T2D) and chronic inflammation in varying degrees. So far, it remains unclear whether the presence of MetS, a cluster of inter-related risk factors for future cardiovascular diseases (CVD) and T2D, is affecting HR-QoL. Some studies suggest that the inflammatory state of a person may be related to HR-QoL [15–17]. However, this relationship has only been studied in elderly men [18] and in patient settings, e.g. among patients with chronic kidney disease or diabetes [15, 16, 19]. We are unaware of any evidence linking HR-QoL to the inflammatory state of individuals with obesity.

In the present study, we analysed data from 13,686 obese men and women from the general Dutch population. The aim of our study is to assess the effect of obesity-related conditions on HR-QoL. These conditions are i) grade of obesity with and without type 2 diabetes (T2D), ii) metabolic syndrome (MetS), and iii) level of inflammation.

## Methods

### Study and participants

We assessed data from the Dutch LifeLines Cohort Study. This multi-disciplinary prospective population-based cohort study examines the health and health-related behaviours of more than 167,000 persons living in the northeast region of the Netherlands [20]. We included participants of Western European origin aged 18–80 years, who enrolled in the study between 2006 and 2012. Participants were excluded if data on clinical and metabolic measurements and data from the questionnaire on HR-QoL were missing or incomplete. In total, 13,686 individuals with obesity (BMI ≥30 kg/m^2^) were included. Prior to the participation in this study, all individuals gave their written informed consent. The study protocol was in accordance with the Declaration of Helsinki and was approved by the medical ethical review committee of the University Medical Center Groningen.

### Measurements and definitions

A standardised protocol was used to obtain blood pressure, height, weight and waist circumference. Blood pressure was measured 10 times during a period of 10 minutes with an automated DINAMAP Monitor (GE Healthcare, Freiburg, Germany). The average of the final three readings was recorded for systolic- and diastolic blood pressure. Anthropometric measurements were done without shoes, for body weight to the nearest 0.1 kg and height and waist circumference to the nearest 0.5 cm. Height was measured with a stadiometer and waist circumference was measured in standing position with a tape measure all around the body, at the level midway between the lower rib margin and the iliac crest.

For analysis of lipids, glucose and the inflammation marker high sensitivity C-reactive protein (hs-CRP), blood samples were drawn in the morning between 8:00 and 10:00 a.m. after a period of overnight fasting. High-density lipoprotein cholesterol was measured with an enzymatic colorimetric method and triglycerides with a colorimetric UV method, on a Roche Modular P chemistry analyser (Roche, Basel, Switzerland). Fasting blood glucose was measured with a hexokinase method. Hs-CRP was determined by nephelometry (BN II system Siemens, Marburg, Germany). The American Heart Association has defined that hs-CRP levels of <1, 1 to 3, and >3 mg/L correspond to low-, moderate-, and high-risk groups for future cardiovascular events. Levels above 10 mg/L usually indicates acute inflammation [21].

BMI was calculated by dividing weight in kilograms by the squared height in meters (kg/m^2^). Based on their BMI, participants were classified into three grades of obesity as defined by the World Health Organisation: obesity grade 1 (BMI 30–34.9); obesity grade 2 (BMI 35.0–39.9); or obesity grade 3 (BMI ≥40.0 kg/m^2^) [22]. MetS was defined according to the modified guidelines of the National Cholesterol Education Program’s Adult Treatment Panel III (NCEP ATP III) [23]. Diagnosis of T2D was based on self-report and/or use of blood-glucose lowering medication, or an elevated fasting blood glucose ≥7.0 mmol/L at examination.

### Measuring HR-QoL using RAND-36

HR-QoL was measured using the RAND 36-Item Health Survey version 1.0 (RAND-36) [24], which was self-completed by the participant. The RAND-36 include the exact same items as the 36-Item Short Form Health Survey version 1.0, however, the scoring for the two domains bodily pain and general health are slightly different. The questionnaire measures health perception across eight multi-item health domains. These include domains mainly related to physical health (i.e. physical functioning, role limitations due to physical health problems, bodily pain and general health) and domains mainly related to mental well-being (i.e. vitality, social functioning, role limitations due to emotional problems, and general mental health). Scores on the eight domains are generated as follows: 1. numeric values are given to each answer for all items, 2. items in the same domain are averaged together to create the eight raw domain scores (the possible combinations of given answers within a domain is fixed, therefore, only fixed scores can be computed), 3. since not all HR-QoL domains include the same amount of questions in the RAND-36, the raw domain scores are transformed to scales of 0 (maximal impairment) to 100 (no impairment) [24]. Please note that the domain scores are not continuous (nor normally distributed), however, based on frequencies of fixed scores.

### Defining morbidities

Participants were asked to indicate in a provided list from which disorders/diseases they currently or in the past suffered. The single morbidities were clustered in the following 11 subgroups: Pulmonary; Cancer; CVD; Head; Gastrointestinal & Liver; Kidney and Bladder; Neurological diseases; Blood disorders; Musculoskeletal diseases; Dermatological diseases; and Mental disorders. If the participant had at least one morbidity within the subgroup, this subgroup was coded as being present. The formation of the subgroups are based on morbidities which affect the same organs or systems, or they share the same biological mechanism. Detailed information on the single morbidities is available in S1 Table.

### Data description and statistical analyses

All analyses were conducted using IBM SPSS Statistics version 20 (IBM Corporation, Armonk, NY, USA). All data are presented for men and women separately. In the characteristics table data are presented as mean ± SD, or median and interquartile range (IQR) when not normally distributed. Unfortunately, the manual of the RAND-36 questionnaire or other publications have not indicated what difference or change in HR-QoL domain scores should be considered as clinically relevant. Therefore, we generated a cut-off value to indicate a poor score based on the HR-QoL domain score at the 25^th^ percentile of the normal weight population. This was done for each domain and separately for men and women. The individuals who were classified as normal weight (BMI ≤25.0 kg/m^2^) also participated in the LifeLines Cohort Study (N = 39,528). To assess whether degree of obesity with and without T2D, MetS and level of inflammation were related to poor scores on the HR-QoL domains, we calculated the percentages of individuals who scored below this arbitrary cut-off value. Chi-square tests were used to analyse the differences in proportions between the groups and Mantel-Haenszel tests to check for a linear trend in proportions across groups.

We performed per HR-QoL domain (outcome measure) three separate multivariable logistic regression analyses to assess the probability of having a poor score according to i) obesity grade with/without T2D, ii) MetS, and iii) level of inflammation. These analyses were sex-stratified and adjusted for age, the 11 subgroups of morbidities, and where appropriate for BMI and T2D. To test whether the associations varied by age, we also tested for a significant interaction between age and the condition under study i) obesity grade with/without T2D, ii) MetS; and iii) level of inflammation). In the model with the exposure variable MetS, T2D was not included as a covariate since diabetes is one of the criteria to define MetS. Since we performed our analysis separately by sex, we accounted for the number of comparisons made within one sex on one domain of HR-QoL (five comparisons within the obesity grade groups with and without T2D, one comparison in the MetS status group and three comparisons within the hs-CRP groups). As such, an arbitrary *P* value < 0.0055 (α = 0.05/9) was considered statistically significant. All *P* values were two-sided.

## Results

### Population characteristics and percentage of obese individuals with a poor HR-QoL

The study population comprised 5,210 (38%) obese men and 8,476 (62%) obese women (Table 1). Mean age was 48 in the men (SD 11 years) and 47 in the women (SD 12 years). In the male population only 3.2% had grade 3 obesity and most participants had hs-CRP levels ≤3 mg/L (68.9%). In the female population we observed a high percentage of individuals with obesity grade 3 (7.9%) and hs-CRP levels above 3 mg/L (58.2%). The prevalence of MetS was 64.1% and 39.9% in respectively, men and women. T2D was present in 9.2% of men and in 6.3% of women.

**Table 1:** Characteristics of participants with obesity.

Characteristics	Men (n = 5,210)	Women (n = 8,476)
Age (yrs)	48 ± 11	47 ± 12
Weight (kg)	108.4 ± 12.6	96.3 ± 12.9
BMI (kg/m^2^)	31.9 (30.7–33.8)	32.9 (31.2–35.6)
Obesity grade 1, n (%)	4363 (83.7)	5961 (70.3)
Obesity grade 2, n (%)	681 (13.1)	1846 (21.8)
Obesity grade 3, n (%)	166 (3.2)	669 (7.9)
Waist circumference (cm)	110.5 (106.5–116.0)	104.0 (98.0–111.0)
Blood glucose (mmol/L)	5.3 (5.0–5.8)	5.1 (4.8–5.5)
Total cholesterol (mmol/L)	5.2 ± 1.0	5.1 ± 1.0
Triglycerides (mmol/L)	1.58 (1.13–2.20)	1.16 (0.85–1.59)
HDL-cholesterol (mmol/L)	1.13 ± 0.26	1.39 ± 0.33
LDL-cholesterol (mmol/L)	3.46 ± 0.91	3.27 ± 0.88
Systolic blood pressure (mmHg)	137 ± 14	129 ± 15
Diastolic blood pressure (mmHg)	80 ± 9	75 ± 9
hs-CRP (mg/L)^**a**^	1.8 (1.0–3.5)	3.8 (1.8–7.1)
<1 mg/L, n (%)	717 (22.6)	502 (9.9)
1–3 mg/L, n (%)	1,468 (46.3)	1,621 (32.0)
3–10 mg/L, n (%)	864 (27.2)	2,337 (46.1)
>10 mg/L, n (%)	124 (3.9)	612 (12.1)
Metabolic syndrome, n (%)	3,339 (64.1)	3,383 (39.9)
Type 2 diabetes, n (%)	480 (9.2)	533 (6.3)

Data presented as mean ± SD or median (interquartile range).

^a^ hs-CRP measures were available in 3,173 men and 5,072 women.

BMI = body mass index; HDL = high-density lipoprotein; LDL = low-density lipoprotein; hs-CRP = high sensitivity C-reactive protein.

**Fig 1:**
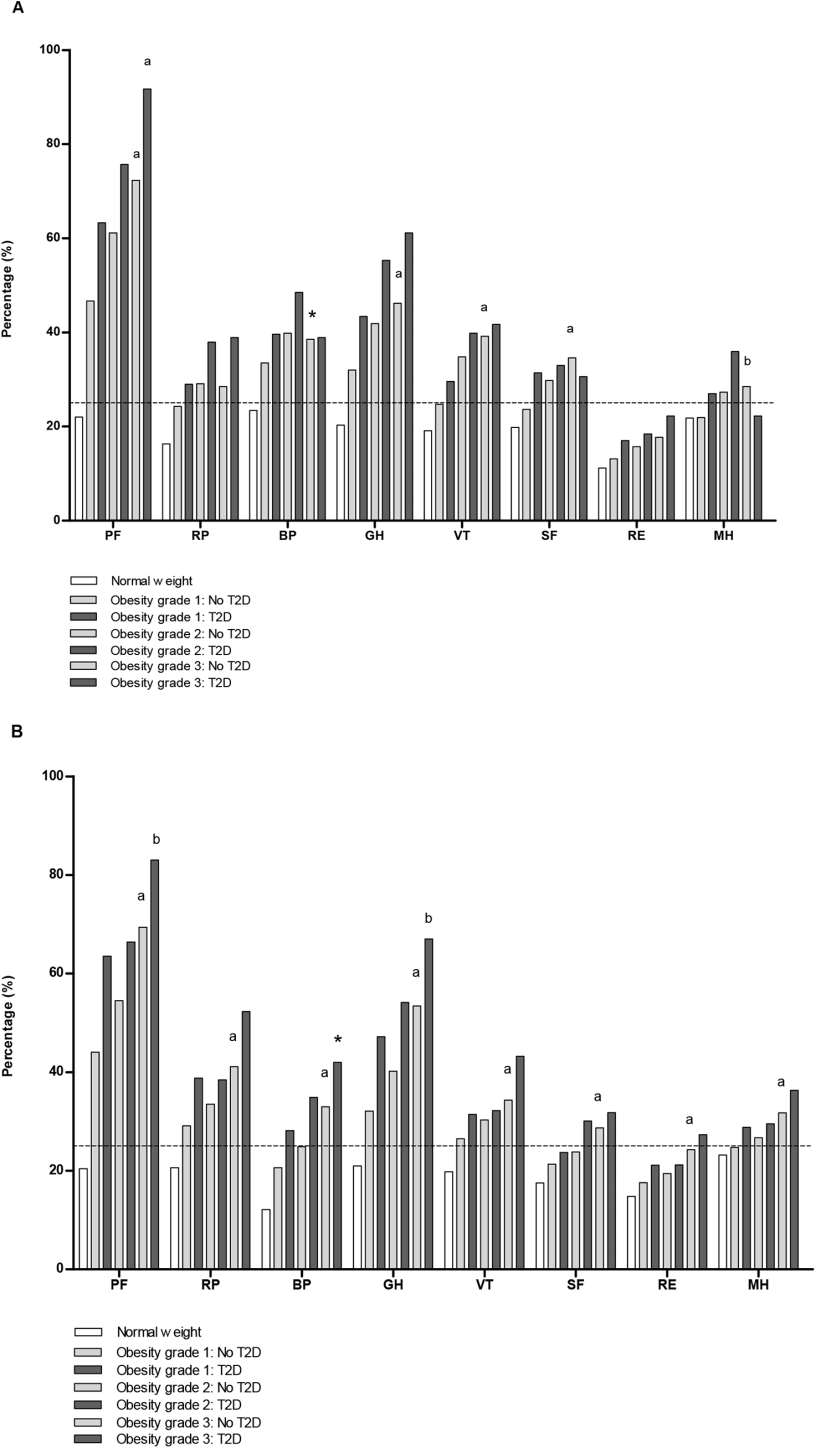
Percentages of obese individuals with a poor HR-QoL domain score, according to obesity grade with and without T2D. (A) Men. (B) Women. Obesity grade 1: BMI 30–34.9 kg/m^2^ Obesity grade 2: BMI 35–39.9 kg/m^2^ Obesity grade 3: BMI ≥40 kg/m^2^ The corresponding cut-off value derived from the scores in the normal weight population for the individual domains (25^th^ percentile men, 25^th^ percentile women): PF = physical functioning (95.0, 90.0); RP = role limitations due to physical health problems (100.0, 100.0); BP = bodily pain (79.6, 67.4); GH = general health (65.0, 65.0); VT = vitality (60.0, 55.0); SF = social functioning (87.5, 75.0); RE = role limitations due to emotional problems (100.0, 100.0); MH = mental health (76.0, 72.0). Mantel-Haenszel tests was used to check for a linear trend in proportions across groups of obesity grade without T2D and separately for groups of obesity grade with T2D. * indicates a linear trend at *P* <0.005; ^**a**^ *P* <0.001; and ^**b**^ *P* <0.002.

**Fig 2:**
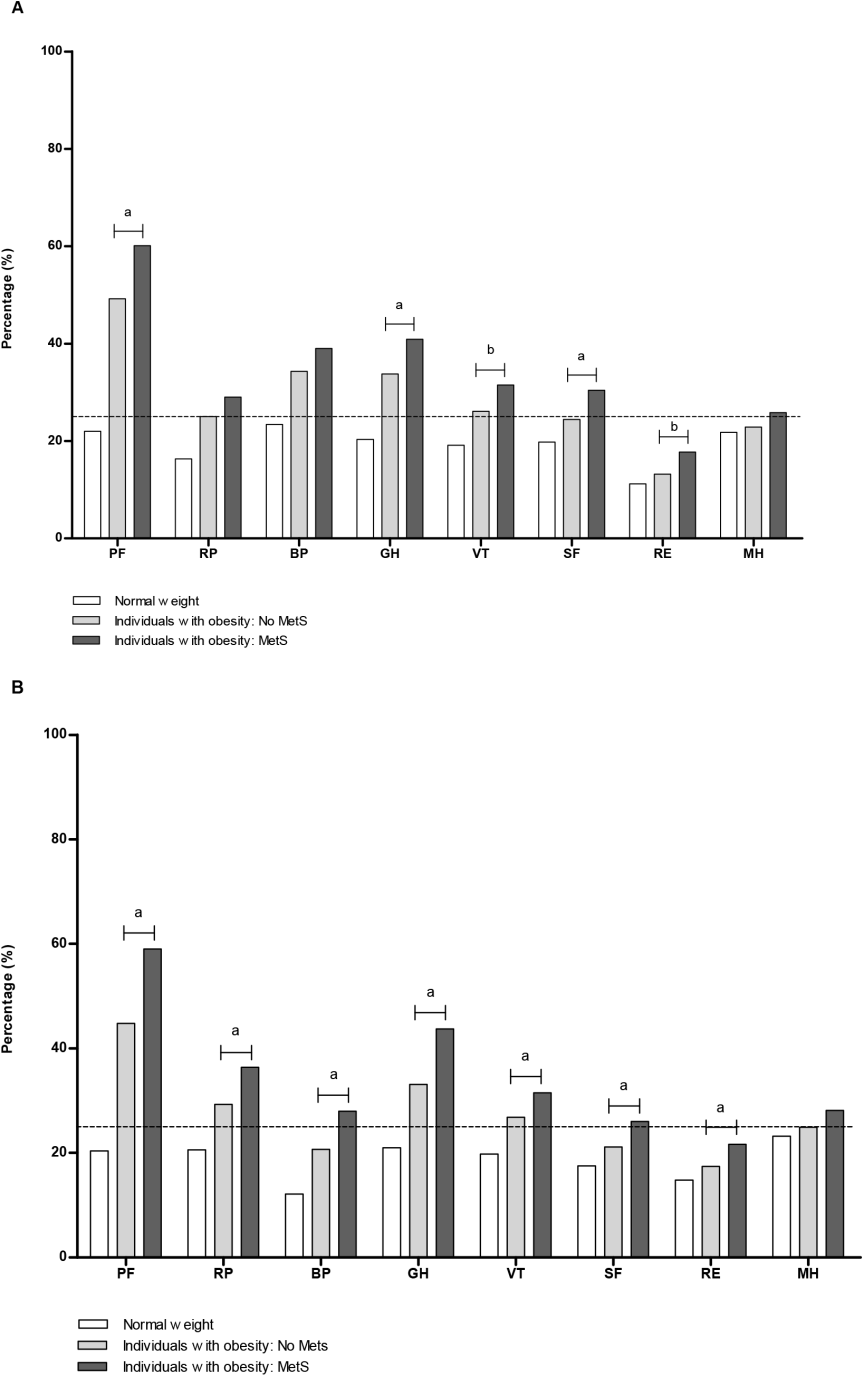
Percentages of obese individuals with a poor HR-QoL domain score, according to MetS. (A) Men. (B) Women. MetS = metabolic syndrome. The corresponding cut-off value derived from the scores in the normal weight population for the individual domains (25^th^ percentile men, 25^th^ percentile women): PF = physical functioning (95.0, 90.0); RP = role limitations due to physical health problems (100.0, 100.0); BP = bodily pain (79.6, 67.4); GH = general health (65.0, 65.0); VT = vitality (60.0, 55.0); SF = social functioning (87.5, 75.0); RE = role limitations due to emotional problems (100.0, 100.0); MH = mental health (76.0, 72.0). Chi-square tests were used to analyse the differences in proportions between the obese without MetS and the obese with MetS. * indicates a difference relative to the reference group (individuals with obesity in the absence of MetS) at *P* <0.005; ^**a**^ *P* <0.001; and ^**b**^ *P* <0.002.

**Fig 3:**
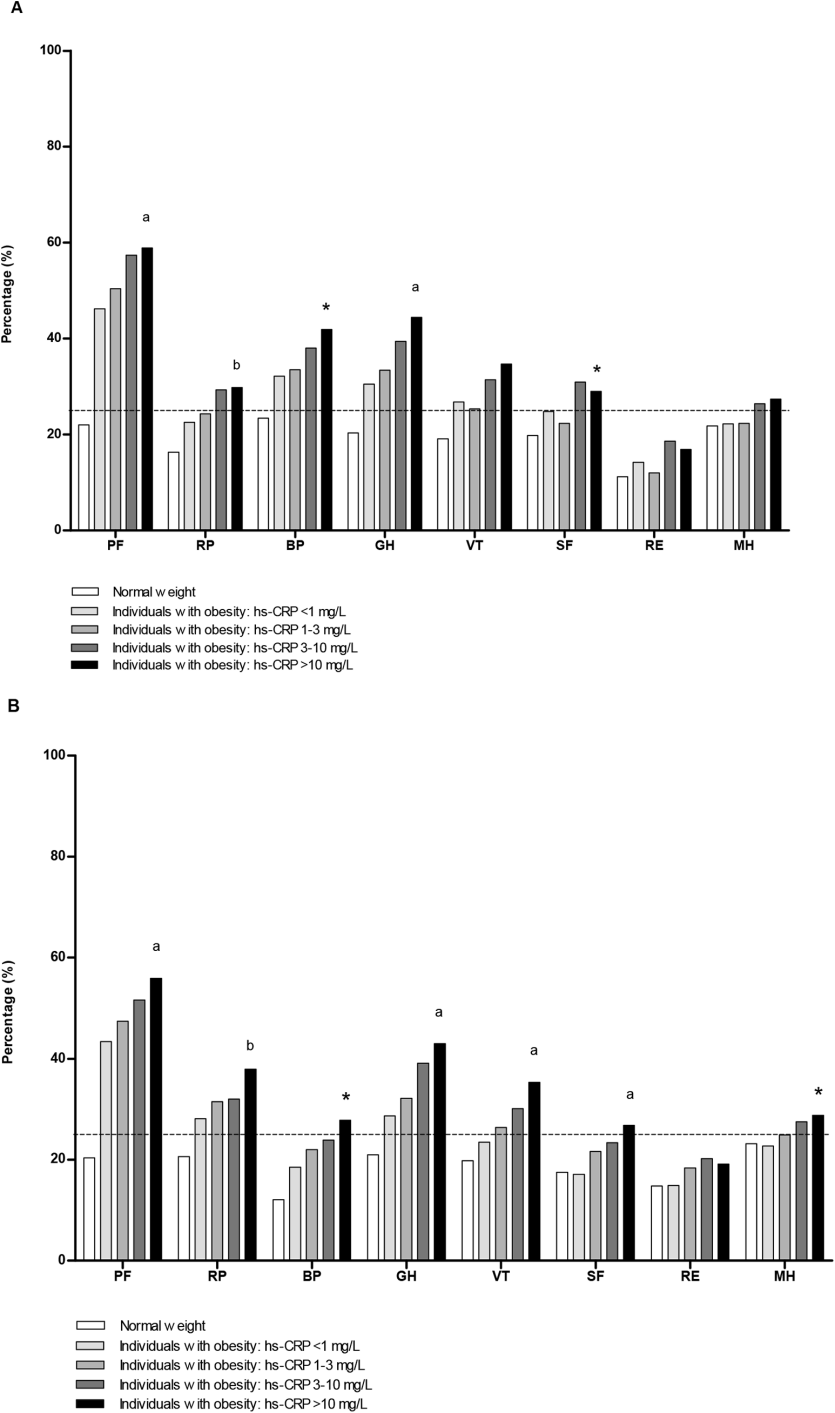
Percentages of obese individuals with a poor HR-QoL domain score, according to level of hs-CRP. (A) Men. (B) Women. hs-CRP = high sensitivity C-reactive protein. The corresponding cut-off value derived from the scores in the normal weight population for the individual domains (25^th^ percentile men, 25^th^ percentile women): PF = physical functioning (95.0, 90.0); RP = role limitations due to physical health problems (100.0, 100.0); BP = bodily pain (79.6, 67.4); GH = general health (65.0, 65.0); VT = vitality (60.0, 55.0); SF = social functioning (87.5, 75.0); RE = role limitations due to emotional problems (100.0, 100.0); MH = mental health (76.0, 72.0). Mantel-Haenszel tests was used to check for a linear trend in proportions across groups of hs-CRP level within the obese population. * indicates a linear trend for individuals with obesity and higher levels of hs-CRP at *P* <0.005; ^**a**^ *P* <0.001; and ^**b**^ *P* <0.002.

Fig 1, Fig 2 and Fig 3 represent the sex-specific percentages of obese individuals with a poor score on the HR-QoL domains. With a higher grade of obesity and/or who had T2D, higher percentages of individuals had a poor HR-QoL, especially in the domains physical functioning (PF) and general health (GH) (Fig 1). Compared to those with grade 1 obesity and no T2D, 45.0% more men and 39.0% more women with grade 3 obesity and T2D had a poor score on physical functioning. For general health these differences were 29.1% and 34.9%, respectively. For all domains, the percentage of men and women with obesity and a poor score was higher in those with MetS (Fig 2). Again, poor scores on HR-QoL was most often reported in the domains physical functioning and general health. Individuals with higher levels of inflammation scored worse on HR-QoL (Fig 3). This trend was most prominent in the group with hs-CRP >3 mg/L, and in the domains mainly related to physical health and the domain vitality (VT). The percentage of individuals who had a poor score on the domain bodily pain varied between 32.2–41.9% in men and 18.5–27.8% in women (Fig 3).

### Probability of having a poor HR-QoL in the obese population

Tables 2–4 show the adjusted odds ratios (ORs) for having a poor HR-QoL score on the eight domains, according to grade of obesity with and without T2D (Table 2), MetS status (Table 3) and level of inflammation (Table 4). There were no significant interactions between age and the conditions under study (data not shown). Compared with the reference group (obesity grade 1 and no T2D), both men and women with grade 2 or 3 obesity and/or T2D had a higher odds ratio for a poor HR-QoL score in the domains physical functioning and general health (*P* <0.001) (Table 2). There were two notable results among men and women with grade 3 obesity. Firstly, the association with poor physical health was very high in men (OR 4.08 (without T2D) and 11.34 (with T2D)) and women (OR 3.39 (without T2D) and 5.05 (with T2D)), with both associations highly significant (*P* <0.0001). Secondly, women with grade 3 obesity, having no T2D, had a greater probability for poor role limitation due to physical health problems (RP) (OR 1.55) and bodily pain (BP) (OR 1.62) compared to the reference group, while men did not. When accompanied by T2D these associations were even stronger (OR 2.24 for RP and OR 2.68 for BP). Although domains of mental well-being were not affected with the same strength, men with obesity grade 2 and 3 without T2D had an OR of, respectively, 1.49 and 2.00 for a poor score on vitality (*P* <0.001) (Table 2).

**Table 2:** Adjusted odds ratios (95% confidence intervals) for having a poor score on each domain of HR-QoL, according to grade of obesity and T2D.

	**Number of subjects (%)**	**Physical Functioning**	**Role limitationsPhysical health**	**Bodily Pain**	**General Health**
**Men**					
Obesity grade 1—no T2D (ref.)	4,022 (77.2)	1.0	1.0	1.0	1.0
Obesity grade 1—T2D	341 (6.5)	**1.48 (1.15–1.89)** ^**b**^	1.13 (0.87–1.47)	1.15 (0.90–1.48)	**1.61 (1.26–2.04)** ^**a**^
Obesity grade 2 –no T2D	578 (11.1)	**1.84 (1.52–2.22)** ^**a**^	1.16 (0.94–1.42)	1.23 (1.02–1.49)	**1.44 (1.19–1.74)** ^**a**^
Obesity grade 2 –T2D	103 (2.0)	**2.79 (1.73–4.49)** ^**a**^	1.55 (1.00–2.40)	1.52 (1.00–2.33)	**2.21 (1.45–3.36)** ^**a**^
Obesity grade 3 –no T2D	130 (2.5)	**4.08 (2.72–6.12)** ^**a**^	1.38 (0.91–2.07)	1.48 (1.01–2.15)	**2.00 (1.39–2.89)** ^**a**^
Obesity grade 3 –T2D	36 (0.7)	**11.34 (3.39–37.89)** ^**a**^	1.60 (0.77–3.29)	0.97 (0.47–2.00)	**3.07 (1.51–6.23)** ^**b**^
**Women**					
Obesity grade 1 –no T2D (ref.)	5,662 (66.8)	1.0	1.0	1.0	1.0
Obesity grade 1 –T2D	299 (3.5)	1.35 (1.02–1.75)	1.31 (1.01–1.70)	1.09 (0.84–1.41)	**1.74 (1.35–2.25)** ^**a**^
Obesity grade 2 –no T2D	1,700 (20.1)	**1.66 (1.47–1.87)** ^**a**^	1.19 (1.05–1.34)	1.14 (1.01–1.29)	**1.36 (1.21–1.54)** ^**a**^
Obesity grade 2 –T2D	146 (1.7)	1.55 (1.05–2.28)	1.10 (0.76–1.59)	1.23 (0.86–1.77)	**2.06 (1.44–2.96)** ^**a**^
Obesity grade 3 –no T2D	581 (6.9)	**3.39 (2.78–4.15)** ^**a**^	**1.55 (1.30–1.87)** ^**a**^	**1.62 (1.34–1.95)** ^**a**^	**2.29 (1.91–2.77)** ^**a**^
Obesity grade 3 –T2D	88 (1.0)	**5.05 (2.77–9.21)** ^**a**^	**2.24 (1.43–3.51)** ^**a**^	**2.68 (1.67–4.29)** ^**a**^	**3.73 (2.32–5.99)** ^**a**^
	**Number of subjects (%)**	**Vitality**	**Social Functioning**	**Role limitations Emotional problems**	**Mental health**
**Men**					
Obesity grade 1—no T2D (ref.)	4,022 (77.2)	1.0	1.0	1.0	1.0
Obesity grade 1—T2D	341 (6.5)	1.43 (1.10–1.86)	**1.53 (1.17–1.99)** ^**b**^	1.35 (0.98–1.87)	1.44 (1.10–1.90)
Obesity grade 2 –no T2D	578 (11.1)	**1.49 (1.22–1.81)** ^**a**^	1.25 (1.01–1.54)	1.09 (0.84–1.41)	1.22 (0.98–1.51)
Obesity grade 2 –T2D	103 (2.0)	**1.85 (1.20–2.85)**	1.39 (0.88–2.19)	1.25 (0.72–2.17)	1.83 (1.16–2.86)
Obesity grade 3 –no T2D	130 (2.5)	**2.00 (1.37–2.91)** ^**a**^	**1.87 (1.27–2.78)** ^**b**^	1.46 (0.90–2.38)	1.41 (0.93–2.13)
Obesity grade 3 –T2D	36 (0.7)	2.10 (1.03–4.28)	1.22 (0.56–2.66)	1.70 (0.71–4.07)	0.87 (0.36–2.10)
**Women**					
Obesity grade 1 –no T2D (ref.)	5,662 (66.8)	1.0	1.0	1.0	1.0
Obesity grade 1 –T2D	299 (3.5)	1.45 (1.11–1.90)	1.16 (0.86–1.57)	1.30 (0.96–1.77)	1.41 (1.07–1.86)
Obesity grade 2 –no T2D	1,700 (20.1)	1.12 (0.99–1.27)	1.08 (0.94–1.24)	1.06 (0.92–1.22)	1.04 (0.91–1.18)
Obesity grade 2 –T2D	146 (1.7)	1.21 (0.83–1.77)	1.37 (0.92–2.02)	1.09 (0.71–1.67)	1.18 (0.80–1.74)
Obesity grade 3 –no T2D	581 (6.9)	1.24 (1.03–1.51)	1.31 (1.07–1.61)	1.35 (1.09–1.68)	1.26 (1.03–1.54)
Obesity grade 3 –T2D	88 (1.0)	1.90 (1.21–3.00)	1.45 (0.89–2.36)	1.57 (0.96–2.59)	1.60 (1.00–2.56)

Adjusted for age and the following morbidities: Pulmonary; Cancer; CVD; Head; Gastrointestinal & Liver; Kidney & Bladder; Neurological diseases

Blood disorders; Musculoskeletal diseases; Dermatological diseases and Mental disorders. Ref.: reference.

Odds ratios in bold indicate *P* <0.005

^**a**^ *P* <0.001

^**b**^ *P* <0.002

**Table 3:** Adjusted odds ratios (95% confidence intervals) for having a poor score on each domain of HR-QoL according to MetS.

	**Number of subjects (%)**	**Physical Functioning**	**Role limitations Physical health**	**Bodily Pain**	**General Health**
**Men**					
No MetS (ref.)	4,420 (84.8)	1.0	1.0	1.0	1.0
MetS	790 (15.2)	1.25 (1.06–1.48)	1.12 (0.93–1.34)	1.12 (0.94–1.33)	**1.27 (1.08–1.50)**
**Women**					
No MetS (ref.)	5,790 (68.3)	1.0	1.0	1.0	1.0
MetS	2,686 (31.7)	1.13 (1.02–1.26)	1.17 (1.05–1.30)	1.14 (1.02–1.27)	**1.35 (1.22–1.51)** ^**a**^
	**Number ofsubjects (%)**	**Vitality**	**Social Functioning**	**Role limitations Emotional problems**	**Mental health**
**Men**					
No MetS (ref.)	4,420 (84.8)	1.0	1.0	1.0	1.0
MetS	790 (15.2)	**1.31 (1.10–1.57)**	**1.31 (1.09–1.57)**	**1.40 (1.12–1.75)**	1.16 (0.96–1.41)
**Women**					
No MetS (ref.)	5,790 (68.3)	1.0	1.0	1.0	1.0
MetS	2,686 (31.7)	**1.27 (1.14–1.42)** ^**a**^	**1.24 (1.10–1.40)** ^**a**^	**1.26 (1.11–1.43)** ^**a**^	1.17 (1.04–1.31)

Adjusted for age, BMI and the following morbidities: Pulmonary; Cancer; CVD; Head; Gastrointestinal & Liver; Kidney & Bladder; Neurological diseases

Blood disorders; Musculoskeletal diseases; Dermatological diseases and Mental disorders. Ref.: reference.

Odds ratios in bold indicate *P* <0.005

^**a**^ *P* <0.001

^**b**^ *P* <0.002

**Table 4:** Adjusted odds ratios (95% confidence intervals) for having a poor score on each domain of HR-QoL according to level of hs-CRP.

	**Number of subjects (%)**	**Physical Functioning**	**Role limitations Physical health**	**Bodily Pain**	**General Health**
**Men**					
hs-CRP <1 mg/L (ref.)	717 (22.6)	1.0	1.0	1.0	1.0
hs-CRP 1–3 mg/L	1,468 (46.3)	1.12 (0.93–1.37)	1.08 (0.86–1.35)	1.04 (0.84–1.27)	1.09 (0.89–1.33)
hs-CRP 3–10 mg/L	864 (27.2)	1.29 (1.04–1.61)	1.31 (1.02–1.67)	1.18 (0.94–1.48)	1.25 (1.00–1.57)
hs-CRP >10 mg/L	124 (3.9)	1.26 (0.83–1.92)	1.32 (0.84–2.08)	1.35 (0.89–2.06)	1.50 (0.99–2.28)
**Women**					
hs-CRP <1 mg/L (ref.)	502 (9.9)	1.0	1.0	1.0	1.0
hs-CRP 1–3 mg/L	1,621 (32.0)	1.13 (0.90–1.42)	1.16 (0.92–1.47)	1.24 (0.99–1.55)	1.16 (0.92–1.46)
hs-CRP 3–10 mg/L	2,337 (46.1)	**1.41 (1.13–1.76)** ^**b**^	1.17 (0.92–1.47)	1.25 (1.00–1.55)	**1.50 (1.19–1.88)** ^**b**^
hs-CRP >10 mg/L	612 (12.1)	**1.85 (1.41–2.44)** ^**a**^	**1.55 (1.17–2.05)** ^**b**^	**1.56 (1.19–2.05)** ^**b**^	**1.65 (1.25–2.18)** ^**a**^
	**Number of subjects (%)**	**Vitality**	**Social Functioning**	**Role limitations Emotional problems**	**Mental health**
**Men**					
hs-CRP <1 mg/L (ref.)	717 (22.6)	1.0	1.0	1.0	1.0
hs-CRP 1–3 mg/L	1,468 (46.3)	0.86 (0.70–1.07)	0.81 (0.65–1.02)	0.75 (0.57–0.99)	0.95 (0.76–1.20)
hs-CRP 3–10 mg/L	864 (27.2)	1.05 (0.83–1.33)	1.17 (0.92–1.48)	1.21 (0.90–1.63)	1.11 (0.86–1.43)
hs-CRP >10 mg/L	124 (3.9)	1.26 (0.82–1.94)	1.07 (0.67–1.69)	1.16 (0.66–2.02)	1.25 (0.78–1.99)
**Women**					
hs-CRP <1 mg/L (ref.)	502 (9.9)	1.0	1.0	1.0	1.0
hs-CRP 1–3 mg/L	1,621 (32.0)	1.19 (0.93–1.52)	1.37 (1.04–1.80)	1.31 (0.99–1.75)	1.17 (0.91–1.51)
hs-CRP 3–10 mg/L	2,337 (46.1)	**1.41 (1.11–1.79)**	**1.50 (1.15–1.96)**	1.47 (1.11–1.96)	1.33 (1.04–1.71)
hs-CRP >10 mg/L	612 (12.1)	**1.70 (1.27–2.26)** ^**a**^	**1.70 (1.23–2.34)** ^**b**^	1.28 (0.91–1.80)	1.32 (0.98–1.78)

Adjusted for age, BMI and the following morbidities: T2D; Pulmonary; Cancer; CVD; Head; Gastrointestinal & Liver; Kidney & Bladder; Neurological diseases

Blood disorders; Musculoskeletal diseases; Dermatological diseases and Mental disorders. Ref.: reference.

Odds ratios in bold indicate *P* <0.005

^**a**^ *P* <0.001

^**b**^ *P* <0.002

For both men and women, the odds ratios for a poor HR-QoL were higher in the presence of MetS for the domains general health, vitality, social functioning and role limitations due to emotional problems (among men all *Ps* <0.005 and among women all *Ps* <0.001) (Table 3).

In women, elevated hs-CRP levels (>3 mg/L) were associated with poor scores on the HR-QoL domains. The associations were especially strong in women with hs-CRP above 10 mg/L for the domains physical functioning (*P* <0.001), role limitations due to emotional problems (*P* <0.002), bodily pain (*P* <0.002), general health (*P* <0.001), vitality (*P* <0.001), and social functioning (*P* <0.002).

Since BMI, T2D and MetS are not completely independent of inflammation level, we also performed an analysis where in the multivariable logistic regression model hs-CRP was modelled together with obesity grade with and without T2D (S2 Table), and also modelled together with MetS (S3 Table).

## Discussion

To the best of our knowledge, this is the first study to report in a very large population with obesity (13,686 individuals) the sex-specific effect of grade of obesity with and without T2D, the effect of MetS and the effect of level of inflammation on HR-QoL.

A systematic review and meta-analysis was published in 2013 on the association of all-cause mortality with overweight and obesity [25]. Results showed that relative to normal weight individuals, only people with grade 2 and 3 obesity have a significantly higher all-cause mortality rate. Studies among individuals with obesity often focus on obesity-related morbidities and mortality, and rarely on quality of life. Rather than simply focusing on the long-term effects of obesity on health and survival, it is also important to understand the influence of obesity and related conditions on an individual’s daily life. We used data of normal weight individuals from the same cohort study to indicate poor scores on eight domains of HR-QoL. The results showed that individuals with grade 1 obesity already had more often a diminished HR-QoL compared to the normal weight individuals. The reduced HR-QoL already visible among individuals with grade 1 obesity seems to be related to their greater BMI. Although, the presence of co-morbidities might also be partly responsible for this observation, earlier studies have shown that a higher BMI is associated with lower HR-QoL [3].

In the obese population the effect of obesity grade and T2D was particularly evident for the domains of physical functioning and general health, even after adjustment for age and morbidities. Since obese individuals carry more weight, and have to deal with a larger body mass, it is not surprising that the daily performance of physical activities is a daunting task for them. As we observed, it becomes even more difficult in the presence of T2D. These results support the previously reported association between T2D and a reduced quality of life, being already evident at the early stage of the disease, especially in relation to the ability to perform physical activities [26].

Obesity often leads to MetS, a cluster of inter-related risk factors for atherosclerosis, CVD and T2D [23]. Results of data on the impact of MetS on HR-QoL are inconsistent. A study conducted in a sample of nondiabetic Iranian adults showed that, only in women, MetS was associated with lower scores on the domains physical functioning, bodily pain and social functioning [27]. However, this study did not address the influence of BMI or obesity.

In contrast, BMI and obesity were a key factor in our study, and we found that even after adjusting for BMI, individuals who had both obesity and MetS were more likely to have a poor score on HR-QoL domains than individuals with obesity alone. Both obese men and women had in the presence of MetS a higher probability for poor scores on the domains general health, vitality, social functioning and role limitations due to emotional problems. Previously, Vetter *et al*. could not establish a relationship between MetS and HR-QoL in individuals with obesity. This may well be accounted for by the small sample size (390 obese participants, 85% female), the study design (inclusion in a weight reduction program) and their use of summary scores for physical health and mental well-being [28]. Tsai et al. also examined HR-QoL among obese individuals aiming for weight reduction [29]. The association between MetS and the summary score of physical HR-QoL was eliminated when adjusted for BMI. This study did also not investigate the association of MetS with the individual domains of HR-QoL. The validity of the summary scores, which are aggregated from the eight health domains, has been the subject of debate [30, 31]. For this reason, the scores from the individual health domains may be more informative than the summary scores.

Levinger et al. reported that in individuals with an increased number of metabolic risk factors a common characteristic is low aerobic fitness and muscle strength, leading to an impaired capacity to perform activities of daily living (ADLs) and impaired quality of life [32]. Resistance training increased the muscle strength and capacity to perform ADLs in individuals with at least two metabolic risk factors and were followed by improvements in the domains physical functioning, general health, and social functioning, despite no changes in body fat content or aerobic power [32]. In a second study from the same research group of Levinger et al., it was found that among a small group of 55 middle-aged adults, women, but not men, with higher numbers of MetS components were more likely to have impaired physical functioning and to experience bodily pain [33]. However, the findings of this second study were unadjusted for BMI. In our study there was also a strong association between MetS and physical functioning, in both obese men (OR [95% CI]: 1.37 [1.16–1.61], *P* <0.001) and obese women (OR 1.31 [1.18–1.46], *P* <0.001), when we did not take BMI into account. Also women experienced more bodily pain (OR 1.22 [1.10–1.35], *P* <0.001). These findings indicate that BMI, rather than MetS, seems to be associated with lower scores on physical functioning and bodily pain.

The conditions under study here–obesity, T2D and MetS–are not only known to be associated with the progression of CVD but are also all characterised by inflammation [34]. Inflammatory factors have been proposed as being part of the mechanism underlying reduced HR-QoL [15–17]. Since the accumulation of intra-abdominal fat is an important risk factor for inflammation, leading to the elevation of circulating hs-CRP, measurement of this widely used marker provides an indication of whether or not inflammation is likely to impair HR-QoL in individuals with obesity [35, 36].

We found that with increasing levels of inflammation there was an increasing number of obese individuals who reported impaired HR-QoL. In our study, women had higher hs-CRP levels than men (*P* <0.0001). Despite this gender difference, women are in general at lower risk for CVD events than men, which is explained by the fact that women have a greater accumulation of subcutaneous fat, while men carry more visceral fat [37]. Nevertheless, we found that obese women with hs-CRP levels above 3 mg/L had a higher probability for poor HR-QoL domain scores, while obese men did not.

Others have also looked at the link between inflammation and HR-QoL. For example, among chronic kidney disease patients with anaemia, only inflammatory markers such as interleukin (IL)-6, IL-8 and tumour necrosis factor (TNF)-α were correlated with some of the HR-QoL domains, but CRP was not [19]. In such group of non-obese patients, lower levels of hs-CRP might not be strong enough for predicting HR-QoL. However, our additional analysis showed that the found association between obesity grade with and without T2D and HR-QoL (S2 Table), and the association between MetS and HR-QoL, is likely to be partially attributable to elevated hs-CRP levels (S3 Table). For example, the OR for poor quality of life in the domain physical functioning was dropped in for individuals with grade 3 obesity and T2D from 11.34 to 7.27 in men and from 5.05 to 3.98 in women when we also included hs-CRP levels in the model. While our data certainly support the hypothesis that hs-CRP is adversely associated with HR-QoL, future studies should also include measurements of other inflammatory markers.

In our study, the impact of obesity on domains of mental well-being was not as strong as that on domains of physical health. Of the four domains mainly related to mental well-being, vitality was more often affected in obese individuals. Previously published studies also suggest that the impact of obesity on mental well-being is weak. One possible explanation for such a relationship is that obesity only affects mental well-being in individuals whose obesity is accompanied by binge eating [38, 39], or diseases, chronic or otherwise [8]. Recently Jagielski et al. reported a high prevalence of psychological co-morbidity, such as symptoms of anxiety (70.3%) and depression (66.2%) among extreme obese individuals (BMI ≥35 kg/m^2^) who sought assistance in weight-management. However, the authors did not find a significant association between these conditions and adiposity. These findings suggest that the treatment-seeking obese individuals may suffer more from the psychological co-morbidities of extreme obesity than the rest of the obese population [40]. In our crude logistic model (adjusted for age only, data not shown), women with grade 3 obesity had higher odds of having a poor score on all four domains related to mental well-being, compared to those with grade 1 obesity (*P* <0.0001). Men with grade 3 obesity only had a poor outcome on vitality (*P* <0.0001). However, after adjusting for the presence of the various morbidities, most associations disappeared, which suggests that the relationship was attributable to the presence of other morbidities or obesity-related conditions. For instance, MetS was especially related to the domains of mental well-being (vitality, social functioning and role limitations due to emotional problems) and elevated hs-CRP levels in women was besides the domains of physical health, also associated with vitality and social functioning.

The main strength of our study is the large, are the well-characterised obese individuals, derived from the general population. Such large sample size provided us with sufficient statistical power to thoroughly investigate all associations between different obesity-related conditions. We even had data on 835 individuals with morbidly obesity. The normal weight individuals from the same cohort study provided us with a large and suitable reference group to define cut-off values of poor HR-QoL. A further strength of our study is the adjustment for a wide range of physical and mental morbidities, which enabled us to examine the effect of obesity and related conditions on HR-QoL as profound as possible.

A limitation of this study is the cross-sectional design. As prospective data collection for the LifeLines Cohort Study is still ongoing, we might conclude about causality in the follow-up studies. A second limitation is that although the RAND-36 has proven to be highly valid for assessing HR-QoL and is a practical tool for epidemiological research, it remains a generic health status questionnaire. Furthermore, it is possible that our results are subject to volunteer bias. Individuals with obesity who elected to participate in the LifeLines Cohort Study may have had a better physical and mental health than those who did not volunteer. Finally, when assessing morbidity, our study relied on data from a self-reported questionnaire, which may have caused under- or over-reporting.

## Conclusions

A substantial portion of obese individuals in the general population experience physical or mental consequences of their weight, which are reflected in the low scores on domains of health-related quality of life. The impact of obesity on an individual’s quality of life is enhanced by grade of obesity, T2D, MetS and inflammation and are mainly related to reduced physical health, such as in the domain of physical functioning and general health). Although mental well-being is less frequently impaired among the general obese population, they still have a higher probability to experience consequences in the related domains.

## Supporting Information

S1 Table Detailed overview of the single morbidities, clustered in 11 subgroups. (DOCX)

S2 Table Adjusted odds ratios (95% confidence intervals) for having a poor score on each domain of HR-QoL, according to obesity grade with/without T2D and hs-CRP. Adjusted for age and the following morbidities: Pulmonary; Cancer; CVD; Head; Gastrointestinal & Liver; Kidney & Bladder; Neurological diseases; Blood disorders; Musculoskeletal diseases; Dermatological diseases and Mental disorders. Ref.: reference. Odds ratios in bold indicate *P* <0.005; ^a^ *P* <0.001; and ^b^ *P* <0.002.
(DOCX)

S3 Table Adjusted odds ratios (95% confidence intervals) for having a poor score on each domain of HR-QoL, according to MetS and hs-CRP. Adjusted for age, BMI and the following morbidities: Pulmonary; Cancer; CVD; Head; Gastrointestinal & Liver; Kidney & Bladder; Neurological diseases; Blood disorders; Musculoskeletal diseases; Dermatological diseases and Mental disorders. Ref.: reference. Odds ratios in bold indicate *P* <0.005; ^a^ *P* <0.001; and ^b^ *P* <0.002.
(DOCX)
