# Supplementary material for: Health-Related Quality of Life in Relation to Obesity Grade, Type 2 Diabetes, Metabolic Syndrome and Inflammation
Source: PLoS One. 2015 Oct 16;10(10):e0140599. doi: 10.1371/journal.pone.0140599 (PMC4608696; doi:10.1371/journal.pone.0140599)
Supplement: S1 Table — (DOCX) [file pone.0140599.s001.docx]

Table S1. Detailed overview of the single morbidities, clustered in 11 subgroups.

| **Subgroup** | **Morbidities** |
| --- | --- |
| Pulmonary | asthma and COPD |
| Cancer |  |
| Cardiovascular disease | myocardial infarction, stroke, heart valve problems, atherosclerosis, thrombosis and pulmonary embolism |
| Head | migraine, cataracts and chronic throat/sinus infections |
| Gastrointestinal & Liver | hepatitis, cirrhosis of the liver, coeliac disease and gallstones |
| Kidney and Bladder | kidney stones, chronic bladder infection and incontinence |
| Neurological diseases | epilepsy, multiple sclerosis, spasticity, Parkinson’s disease and dementia |
| Blood disorders | anaemia and clotting disorders |
| Musculoskeletal diseases | fibromyalgia, arthrosis, rheumatic disease, osteoporosis, back or neck hernia, and repetitive strain injury |
| Dermatological diseases | serious acne, eczema and psoriasis |
| Mental disorders | chronic fatigue syndrome, burnout, depression, panic disorder, social phobia, agoraphobia, other anxiety syndromes, manic depressive syndrome, schizophrenia, eating problems, obsessive/compulsive disorders and ADHD |
