# Supplementary material for: Health-Related Quality of Life in Relation to Obesity Grade, Type 2 Diabetes, Metabolic Syndrome and Inflammation
Source: PLoS One. 2015 Oct 16;10(10):e0140599. doi: 10.1371/journal.pone.0140599 (PMC4608696; doi:10.1371/journal.pone.0140599)
Supplement: S2 Table — Adjusted for age and the following morbidities: Pulmonary; Cancer; CVD; Head; Gastrointestinal & Liver; Kidney & Bladder; Neurological diseases; Blood disorders; Musculoskeletal diseases; Dermatological diseases and Mental disorders. Ref.: reference. Odds ratios in bold indicate P <0.005; a P <0.001; and b P <0.002. (DOCX) [file pone.0140599.s002.docx]

Table S2. Adjusted odds ratios (95% confidence intervals) for having a poor score on each domain of HR-QoL, according to obesity grade with/without T2D, and hs-CRP.

|  | **Number of subjects (%)** | **Physical**  **Functioning** | **Role limitations**  **Physical health** | **Bodily Pain** | **General Health** |
| --- | --- | --- | --- | --- | --- |
| **Men** |  |  |  |  |  |
| Obesity grade 1 - no T2D (ref.) | 4,022 (77.2) | 1.0 | 1.0 | 1.0 | 1.0 |
| Obesity grade 1 - T2D | 341 (6.5) | **1.59 (1.16-2.18)** | 1.08 (0.77-1.51) | 1.27 (0.93-1.74) | 1.53 (1.13-2.09) |
| Obesity grade 2 - no T2D | 578 (11.1) | **1.82 (1.43-2.33) ^a^** | 0.97 (0.74-1.27) | 1.11 (0.87-1.43) | 1.31 (1.03-1.66) |
| Obesity grade 2 - T2D | 103 (2.0) | 1.79 (1.02-3.15) | 1.38 (0.80-2.37) | 1.18 (0.70-2.01) | **2.16 (1.29-3.64)** |
| Obesity grade 3 - no T2D | 130 (2.5) | **3.70 (2.16-6.34) ^a^** | 1.04 (0.59-1.82) | 1.26 (0.76-2.09) | 1.58 (0.96-2.58) |
| Obesity grade 3 - T2D | 36 (0.7) | 7.27 (1.61-32.80) | 1.78 (0.72-4.40) | 0.74 (0.29-1.87) | 2.83 (1.09-7.37) |
| **Women** |  |  |  |  |  |
| Obesity grade 1 - no T2D (ref.) | 5,662 (66.8) | 1.0 | 1.0 | 1.0 | 1.0 |
| Obesity grade 1 - T2D | 299 (3.5) | **1.82 (1.26-2.64) ^b^** | 1.50 (1.07-2.09) | 1.14 (0.82-1.60) | **1.76 (1.26-2.45) ^b^** |
| Obesity grade 2 - no T2D | 1,700 (20.1) | **1.53 (1.31-1.79) ^a^** | 1.02 (0.87-1.20) | 1.00 (0.85-1.17) | 1.21 (1.04-1.42) |
| Obesity grade 2 - T2D | 146 (1.7) | 1.81 (1.08-3.07) | 1.21 (0.76-1.90) | 1.17 (0.74-1.86) | **2.17 (1.37-3.44) ^b^** |
| Obesity grade 3 - no T2D | 581 (6.9) | **2.66 (2.05-3.45) ^a^** | 1.38 (1.07-1.76) | 1.28 (1.00-1.64) | **1.98 (1.55-2.53) ^a^** |
| Obesity grade 3 - T2D | 88 (1.0) | **3.98 (1.99-7.98) ^a^** | 1.65 (0.96-2.85) | **3.10 (1.72-5.56) ^a^** | **2.90 (1.65-5.10) ^a^** |
|  |  |  |  |  |  |
| **Men** |  |  |  |  |  |
| hs-CRP <1 mg/L (ref.) | 717 (22.6) | 1.0 | 1.0 | 1.0 | 1.0 |
| hs-CRP 1-3 mg/L | 1,468 (46.3) | 1.16 (0.96-1.41) | 1.08 (0.87-1.35) | 1.04 (0.85-1.28) | 1.11 (0.90-1.36) |
| hs-CRP 3-10 mg/L | 864 (27.2) | 1.36 (1.09-1.69) | 1.32 (1.04-1.68) | 1.19 (0.95-1.50) | 1.30 (1.04-1.63) |
| hs-CRP >10 mg/L | 124 (3.9) | 1.33 (0.87-2.03) | 1.33 (0.85-2.09) | 1.38 (0.91-2.11) | 1.56 (1.03-2.36) |
| **Women** |  |  |  |  |  |
| hs-CRP <1 mg/L (ref.) | 502 (9.9) | 1.0 | 1.0 | 1.0 | 1.0 |
| hs-CRP 1-3 mg/L | 1,621 (32.0) | 1.15 (0.92-1.45) | 1.18 (0.93-1.49) | 1.26 (1.00-1.57) | 1.17 (0.92-1.48) |
| hs-CRP 3-10 mg/L | 2,337 (46.1) | **1.47 (1.18-1.83) ^b^** | 1.19 (0.95-1.50) | 1.28 (1.03-1.60) | **1.52 (1.21-1.91) ^a^** |
| hs-CRP >10 mg/L | 612 (12.1) | **1.98 (1.51-2.61) ^a^** | **1.60 (1.21-2.11) ^b^** | **1.62 (1.23-2.12) ^a^** | **1.70 (1.29-2.24) ^a^** |

Adjusted for age and the following morbidities: Pulmonary; Cancer; CVD; Head; Gastrointestinal & Liver; Kidney & Bladder; Neurological diseases;
Blood disorders; Musculoskeletal diseases; Dermatological diseases and Mental disorders. Ref.: reference.
Odds ratios in bold indicate *P* <0.005; **^a^** *P* <0.001; and **^b^** *P* <0.002.

Table S2. Adjusted odds ratios (95% confidence intervals) for having a poor score on each domain of HR-QoL, according to obesity grade with/without T2D and hs-CRP.

| ***Continued*** | **Number of subjects (%)** | **Vitality** | **Social**  **Functioning** | **Role limitations**  **Emotional problems** | **Mental Health** |
| --- | --- | --- | --- | --- | --- |
| **Men** |  |  |  |  |  |
| Obesity grade 1 - no T2D (ref.) | 4,022 (77.2) | 1.0 | 1.0 | 1.0 | 1.0 |
| Obesity grade 1 - T2D | 341 (6.5) | 1.48 (1.06-2.07) | **1.64 (1.18-2.29)** | 1.47 (0.98-2.19) | 1.51 (1.07-2.13) |
| Obesity grade 2 - no T2D | 578 (11.1) | 1.38 (1.07-1.77) | 1.03 (0.79-1.35) | 0.98 (0.71-1.37) | 1.12 (0.86-1.47) |
| Obesity grade 2 - T2D | 103 (2.0) | 1.61 (0.94-2.75) | 1.08 (0.60-1.92) | 1.07 (0.53-2.14) | 1.56 (0.89-2.75) |
| Obesity grade 3 - no T2D | 130 (2.5) | 1.78 (1.08-2.94) | 1.70 (1.01-2.86) | 0.99 (0.49-1.99) | 1.13 (0.64-1.99) |
| Obesity grade 3 - T2D | 36 (0.7) | **3.23 (1.30-8.06) ^a^** | 1.64 (0.65-4.16) | 1.41 (0.48-4.10) | 0.79 (0.28-2.29) |
| **Women** |  |  |  |  |  |
| Obesity grade 1 - no T2D (ref.) | 5,662 (66.8) | 1.0 | 1.0 | 1.0 | 1.0 |
| Obesity grade 1 - T2D | 299 (3.5) | **1.73 (1.23-2.44) ^b^** | 1.46 (1.01-2.12) | 1.44 (0.98-2.10) | 1.65 (1.16-2.35) |
| Obesity grade 2 - no T2D | 1,700 (20.1) | 1.09 (0.92-1.28) | 1.08 (0.90-1.29) | 0.98 (0.81-1.19) | 1.08 (0.91-1.28) |
| Obesity grade 2 - T2D | 146 (1.7) | 1.40 (0.88-2.24) | 1.43 (0.87-2.33) | 1.12 (0.67-1.89) | 1.46 (0.91-2.36) |
| Obesity grade 3 - no T2D | 581 (6.9) | 1.13 (0.88-1.46) | 1.30 (0.99-1.71) | **1.51 (1.15-1.99)** | 1.20 (0.92-1.56) |
| Obesity grade 3 - T2D | 88 (1.0) | 1.52 (0.87-2.65) | 1.36 (0.75-2.44) | 1.65 (0.92-2.95) | 1.42 (0.81-2.52) |
|  |  |  |  |  |  |
| **Men** |  |  |  |  |  |
| Hs-CRP <1 mg/L (ref.) | 717 (22.6) | 1.0 | 1.0 | 1.0 | 1.0 |
| Hs-CRP 1-3 mg/L | 1,468 (46.3) | 0.87 (0.71-1.08) | 0.83 (0.67-1.04) | 0.75 (0.57-1.00) | 0.96 (0.76-1.21) |
| Hs-CRP 3-10 mg/L | 864 (27.2) | 1.08 (0.85-1.36) | 1.22 (0.95-1.55) | 1.22 (0.91-1.63) | 1.14 (0.88-1.46) |
| Hs-CRP >10 mg/L | 124 (3.9) | 1.29 (0.84-1.98) | 1.12 (0.71-1.77) | 1.17 (0.67-2.03) | 1.28 (0.80-2.03) |
| **Women** |  |  |  |  |  |
| Hs-CRP <1 mg/L (ref.) | 502 (9.9) | 1.0 | 1.0 | 1.0 | 1.0 |
| Hs-CRP 1-3 mg/L | 1,621 (32.0) | 1.19 (0.93-1.53) | 1.37 (1.04-1.81) | 1.32 (0.99-1.76) | 1.17 (0.91-1.50) |
| Hs-CRP 3-10 mg/L | 2,337 (46.1) | **1.42 (1.11-1.80)** | **1.51 (1.15-1.97)** | 1.49 (1.13-1.97) | 1.32 (1.04-1.69) |
| Hs-CRP >10 mg/L | 612 (12.1) | **1.72 (1.29-2.29) ^a^** | **1.72 (1.25-2.37) ^b^** | 1.29 (0.91-1.81) | 1.31 (0.98-1.78) |

Adjusted for age, BMI and the following morbidities: Pulmonary; Cancer; CVD; Head; Gastrointestinal & Liver; Kidney & Bladder; Neurological diseases;
Blood disorders; Musculoskeletal diseases; Dermatological diseases and Mental disorders. Ref.: reference.
Odds ratios in bold indicate *P* <0.005; **^a^** *P* <0.001; and **^b^** *P* <0.002.
