# Supplementary material for: Health-Related Quality of Life in Relation to Obesity Grade, Type 2 Diabetes, Metabolic Syndrome and Inflammation
Source: PLoS One. 2015 Oct 16;10(10):e0140599. doi: 10.1371/journal.pone.0140599 (PMC4608696; doi:10.1371/journal.pone.0140599)
Supplement: S3 Table — Adjusted for age, BMI and the following morbidities: Pulmonary; Cancer; CVD; Head; Gastrointestinal & Liver; Kidney & Bladder; Neurological diseases; Blood disorders; Musculoskeletal diseases; Dermatological diseases and Mental disorders. Ref.: reference. Odds ratios in bold indicate P <0.005; a P <0.001; and b P <0.002. (DOCX) [file pone.0140599.s003.docx]

Table S3. Adjusted odds ratios (95% confidence intervals) for having a poor score on each domain of HR-QoL, according to MetS and hs-CRP.

|  | **Number of subjects (%)** | **Physical**  **Functioning** | **Role limitations**  **Physical health** | **Bodily Pain** | **General Health** |
| --- | --- | --- | --- | --- | --- |
| **Men** |  |  |  |  |  |
| No MetS (ref.) | 4,420 (84.8) | 1.0 | 1.0 | 1.0 | 1.0 |
| MetS | 790 (15.2) | 1.22 (0.99-1.51) | 1.05 (0.84-1.32) | 1.11 (0.90-1.37) | 1.26 (1.03-1.56) |
| **Women** |  |  |  |  |  |
| No MetS (ref.) | 5,790 (68.3) | 1.0 | 1.0 | 1.0 | 1.0 |
| MetS | 2,686 (31.7) | 1.09 (0.95-1.25) | 1.16 (1.01-1.33) | 1.11 (0.99-1.01) | **1.30 (1.13-1.49) ^a^** |
|  |  |  |  |  |  |
| **Men** |  |  |  |  |  |
| hs-CRP <1 mg/L (ref.) | 717 (22.6) | 1.0 | 1.0 | 1.0 | 1.0 |
| hs-CRP 1-3 mg/L | 1,468 (46.3) | 1.12 (0.92-1.36) | 1.08 (0.86-1.35) | 1.03 (0.84-1.26) | 1.08 (0.88-1.32) |
| hs-CRP 3-10 mg/L | 864 (27.2) | 1.29 (1.03-1.61) | 1.31 (1.02-1.67) | 1.18 (0.94-1.48) | 1.25 (1.00-1.57) |
| hs-CRP >10 mg/L | 124 (3.9) | 1.27 (0.83-1.94) | 1.34 (0.85-2.10) | 1.36 (0.89-2.07) | 1.53 (1.01-2.32) |
| **Women** |  |  |  |  |  |
| hs-CRP <1 mg/L (ref.) | 502 (9.9) | 1.0 | 1.0 | 1.0 | 1.0 |
| hs-CRP 1-3 mg/L | 1,621 (32.0) | 1.12 (0.90-1.41) | 1.16 (0.92-1.46) | 1.23 (0.98-1.55) | 1.14 (0.90-1.45) |
| hs-CRP 3-10 mg/L | 2,337 (46.1) | **1.41 (1.13-1.75)** | 1.16 (0.92-1.45) | 1.24 (0.99-1.54) | **1.47 (1.17-1.84) ^b^** |
| hs-CRP >10 mg/L | 612 (12.1) | **1.85 (1.40-2.43) ^a^** | **1.53 (1.16-2.03)** | **1.55 (1.18-2.04) ^b^** | **1.61 (1.22-2.13) ^b^** |

Adjusted for age, BMI and the following morbidities: Pulmonary; Cancer; CVD; Head; Gastrointestinal & Liver; Kidney & Bladder; Neurological diseases;
Blood disorders; Musculoskeletal diseases; Dermatological diseases and Mental disorders. Ref.: reference.
Odds ratios in bold indicate *P* <0.005; **^a^** *P* <0.001; and **^b^** *P* <0.002.

Table S3. Adjusted odds ratios (95% confidence intervals) for having a poor score on each domain of HR-QoL, according to MetS and hs-CRP.

| *Continued* | Number of subjects (%) | Vitality | Social  Functioning | Role limitations  Emotional problems | Mental Health |
| --- | --- | --- | --- | --- | --- |
| Men |  |  |  |  |  |
| No MetS (ref.) | 4,420 (84.8) | 1.0 | 1.0 | 1.0 | 1.0 |
| MetS | 790 (15.2) | 1.26 (1.01-1.57) | 1.21 (0.96-1.52) | 1.24 (0.94-1.64) | 1.10 (0.87-1.40) |
| Women |  |  |  |  |  |
| No MetS (ref.) | 5,790 (68.3) | 1.0 | 1.0 | 1.0 | 1.0 |
| MetS | 2,686 (31.7) | **1.28 (1.11-1.47) ^b^** | **1.27 (1.09-1.49)** | **1.22 (0.99-1.01) ^a^** | 1.19 (1.02-1.38) |
|  |  |  |  |  |  |
| Men |  |  |  |  |  |
| hs-CRP <1 mg/L (ref.) | 717 (22.6) | 1.0 | 1.0 | 1.0 | 1.0 |
| hs-CRP 1-3 mg/L | 1,468 (46.3) | 0.85 (0.69-1.06) | 0.81 (0.64-1.01) | 0.74 (0.56-0.99) | 0.94 (0.75-1.19) |
| hs-CRP 3-10 mg/L | 864 (27.2) | 1.05 (0.83-1.33) | 1.17 (0.92-1.49) | 1.21 (0.90-1.62) | 1.11 (0.86-1.43) |
| hs-CRP >10 mg/L | 124 (3.9) | 1.28 (0.83-1.97) | 1.09 (0.69-1.72) | 1.17 (0.67-2.04) | 1.27 (0.80-2.03) |
| Women |  |  |  |  |  |
| hs-CRP <1 mg/L (ref.) | 502 (9.9) | 1.0 | 1.0 | 1.0 | 1.0 |
| hs-CRP 1-3 mg/L | 1,621 (32.0) | 1.18 (0.92-1.51) | 1.36 (1.03-1.79) | 1.31 (0.98-1.74) | 1.16 (0.90-1.50) |
| hs-CRP 3-10 mg/L | 2,337 (46.1) | 1.38 (1.09-1.76) | **1.47 (1.13-1.93)** | 1.45 (1.10-1.92) | 1.32 (1.03-1.69) |
| hs-CRP >10 mg/L | 612 (12.1) | **1.66 (1.24-2.22) ^b^** | **1.66 (1.20-2.29)** | 1.25 (0.89-1.77) | 1.31 (0.97-1.77) |

Adjusted for age, BMI and the following morbidities: Pulmonary; Cancer; CVD; Head; Gastrointestinal & Liver; Kidney & Bladder; Neurological diseases;
Blood disorders; Musculoskeletal diseases; Dermatological diseases and Mental disorders. Ref.: reference.
Odds ratios in bold indicate *P* <0.005; **^a^** *P* <0.001; and **^b^** *P* <0.002.
